# The Book World of Medicine and Science

**Published:** 1898-06-11

**Authors:** 


### The Book World of Medicine and Science.

Report on Bubonic Plague. By Khan Bahadur N. H. Choksy. (Bombay, 1897. By Authority.) Pp. 55, 4to.

Khan Bahadur Choksy, the writer of this lucid and admirable report, has been in medical charge, during the recent epidemics, of the Arthur Road Hospital at Bombay. The report is based on observations of 939 cases of plague seen by him between September, 1896, and February, 1897, and is the result, therefore, of much laborious clinical and pathological work. Of the cases observed by Khan Bahadur Choksy, fully two-thirds were low caste Hindus, and the remainder chiefly Mussulmen and Goanese. As might be expected, the recovery rate was highest amongst the better classes, while, from another point of view, it appeared that the death-rate was lower amongst children and the less exclusively vegetarian cases. Khan Bahadur Choksy recognises five main varieties or types of bubonic plague: Pestis minor, P. ambulans, P. simplex, P. septica, and P. pulmonalis. P. minor and P. ambulans are self-explanatory terms; P. simplex indicates those cases in which the specific bacillary injection is restricted to the lymphatic system, and P. septica is the term employed when the bacilli are found in the blood. P. pulmonalis is not merely plague with pneumonia, but a definite variety analogous to the pulmonary forms of anthrax met with amongst wool-sorters. Indeed, in more than one way, the natural history of plague seems very similar to that of anthrax. In treating plague Khan Bahadur Choksy attaches considerable importance to the free use of digitalis and alcohol, and the injection of the buboes with antiseptic fluids. It is interesting to note that calcium chloride has a decided influence in limiting infiltration, and that serum-therapy (unless it be that of Professor Lustig) is—as a curative method—but a broken reed. Hæmorrhages, in every organ and every situation, seem to have been the most notable feature in Khan Bahadur's necropsies. This report is, on the whole, an exceedingly valuable one. It is a storehouse of observed facts carefully recorded, and deserves the attention of every systematist.

Consumption: How to Avoid It, and Weak Eyes. By B. Schwarzbach, M.D. (London: Digby, Long, and Co. 1897. Pp. 107. Price 2s. 6d.)

The ambiguity of the title of this book is in pleasing contrast with the very definite purpose of its publication. The subject matter consists of the text of two lectures, originally delivered in Australia, under the auspices (we are told) of His Worship the Mayor of Sydney and His Excellency the Acting Governor of New South Wales. These lectures have been published, the author informs us, since he has taken up residence in London, at the kind insistence of many former patients, and with a "still nobler aim than curing diseases—preventing their appearance." We fear that His Worship and His Excellency had to listen to a great deal that was commonplace, a great deal that was platitudinous, and not a few syntactical eccentricities.

Some Incidents in General Practice. By Augustin Prichard. (Bristol: J. W. Arrowsmith. 1898. Price 2s. 6d. Pp. 93. Two illustrations.)

This little volume is a pleasantly discursive collection of reminiscences, mostly anecdotal, from the pen of the late Mr. Augustin Prichard. It throws many sidelights, humorous and pathetic, on the daily round of a general practitioner: the little weaknesses and foibles of human nature, as the doctor sees it, are touched on kindly and gently; the pathos of suffering, with true and deep feeling. These memories are the memories of a kindly, large-hearted, and genial man, written in the evening of his days with the consciousness of life's work well done. They will doubtless give pleasure to not a few, and can give pain to none. The excellent craftsmanship of Bristol printers and bookbinders is well known, and is worthily exemplified by this little volume from Messrs. Arrowsmith's press.

Vade Mecum of Ophthalmological Therapeutics. By Dr. E. Landolt, of Paris, and Dr. P. Gygax, of Milwaukee; translated by Dr. E. Neyman, of Milwaukee. (London and Philadelphia: J. B. Lippincott Company. 1898. Price 3s. 6d.)

This is a handy little volume, giving in dictionary form a series of short memoranda as to the treatment of the various diseases and accidents which fall into the hands of the ophthalmologist. Diseases and remedies, together with details as to the use of the various drugs and the application of the various modes of treatment suggested, follow one another just as the requirements of the alphabet demand, and there are plenty of cross references, by means of which the utility of the book is much increased. It is not at first sight apparent to what class of readers this work appeals, for it would be of but little use to anyone who did not possess a fair working knowledge of eye diseases, while to those who do possess such knowledge the suggestions it contains would appear almost superfluous. The two classes who will probably find good in this book are, first, students who, having learnt their eyes practically in the wards and among the out-patients, want a note-book full of hints and formulæ, as this is, rather than a complete treatise; and, secondly, practitioners who wish to have at hand a *résumé* of modern treatment and modern therapeutic methods. On the whole, the treatment recommended is judicious and well represents the teaching of the school from which the book emanates. Here and there remedies are mentioned which will not be familiar to the English reader; but this is not a matter of any great importance. As a memorandum book containing a great deal of information of much the same kind as an advanced student would be likely to put down in his notes, all systematised and arranged for ready reference in alphabetical order, this little work may be highly recommended.

